# Dietary Proportions of Carbohydrates, Fat, and Protein and Risk of Oesophageal Cancer by Histological Type

**DOI:** 10.1371/journal.pone.0054913

**Published:** 2013-01-22

**Authors:** Katarina Lagergren, Anna Lindam, Jesper Lagergren

**Affiliations:** 1 Upper Gastrointestinal Research, Department of Molecular Medicine and Surgery, Karolinska Institutet, Stockholm, Sweden; 2 Division of Cancer Studies, King's College London, London, United Kingdom; Foundation for Liver Research, United Kingdom

## Abstract

**Background:**

Dietary habits influence the risk of cancer of the oesophagus and oesophago-gastric junction, but the role of proportions of the main dietary macronutrients carbohydrates, fats and proteins is uncertain.

**Methods:**

Data was derived from a nationwide Swedish population-based case-control study conducted in 1995–1997, in which case ascertainment was rapid, and all cases were uniformly classified. Information on the subjects' history of dietary intake was collected in personal interviews. Odds ratios (ORs) and 95% confidence intervals (CIs) were calculated using logistic regression, with adjustment for potentially confounding factors.

**Results:**

Included were 189 oesophageal adenocarcinomas, 262 oesophago-gastric adenocarcinomas, 167 oesophageal squamous cell carcinomas, and 820 control subjects. Regarding oesophageal or oesophago-gastric junctional adenocarcinoma, a high dietary proportion of carbohydrates decreased the risk (OR 0.50, CI 0.34–0.73), and a high portion of fat increased the risk (OR 1.96, CI 1.34–2.87), while a high proportion of protein did not influence the risk (OR 1. 08, 95% CI 0.75–1.56). Regarding oesophageal squamous cell carcinoma, the single macronutrients did not influence the risk statistically significantly.

**Conclusions:**

A diet with a low proportion of carbohydrates and a high proportion of fat might increase the risk of oesophageal adenocarcinoma.

## Introduction

Oesophageal cancer is characterised by a poor prognosis (the 5-year survival rate is less than 10%) and a high incidence (the 8^th^ most common cancer globally) [1]. Squamous cell carcinoma is the dominating histological type in non-industrialised countries, but its incidence has decreased in industrialised countries [2]. The incidence of adenocarcinoma of the oesophageal and oesophago-gastric junction, on the other hand, has increased rapidly in Western populations, [3] and currently adenocarcinoma is more common than squamous cell carcinoma in several industrialised populations, particularly in white men [2]. The main risk factors for oesophageal squamous cell carcinoma in industrialised countries are tobacco smoking and alcohol abuse, while adenocarcinoma is mainly associated with gastro-oesophageal reflux and obesity [4]. Moreover, dietary habits have been associated with risk of both histological types of oesophageal cancer. A high dietary intake of fruit (antioxidants), vegetables and fibre is associated with a decreased risk of adenocarcinoma and squamous cell carcinoma of the oesophagus, while a diet high in fat and meats, particularly red meats, seems to be associated with an increased risk of these tumours [5]–[9]. Several other dietary items and patterns have been found to possibly influence the risk of oesophageal cancer, but the current evidence is more limited. No study has addressed how the proportions of the main dietary nutrients, i.e. carbohydrates, protein, and fat, influence the risk of these tumours. Therefore, this study aimed to reveal associations between the proportions of the three main dietary nutrients and the risk of developing oesophageal cancer by histological type.

## Methods

### Ethics statement

All participants provided both written and verbal informed consent to participate in this study. The study was approved by all six regional ethics committees in Sweden, i.e. The Regional Ethical Review Board in Stockholm, The Regional Ethical Review Board in Uppsala, The Regional Ethical Review Board in Umeå, The Regional Ethical Review Board in Linköping, The Regional Ethical Review Board in Göteborg and The Regional Ethical Review Board in Lund.

### Study design

The design and organisation of this population-based and nationwide Swedish case-control study have been described in detail elsewhere, [10] and the aetiological roles of reflux, body mass, tobacco smoking, alcohol consumption, and infection with Helicobacter pylori for oesophageal and oesophago-gastric junctional cancer have been evaluated in this case-control study [10]–[13]. In brief, cases and controls were prospectively included and data collected in 1995 to 1997. The study base was the entire Swedish population aged below 80 years. All newly diagnosed cases of adenocarcinoma of the oesophagus and oesophago-gastric junction, and half of the cases of squamous cell carcinoma of the oesophagus (those born on even numbered dates) were eligible as cases. The reason for including only half of the squamous cell carcinoma cases was that this cancer type was more common in Sweden during the study period, and the main focus of the study was on adenocarcinoma. Population-based control subjects were randomly selected from 10-year age and gender strata in the entire Swedish population. The controls were frequency-matched regarding age and sex to the cases of oesophageal adenocarcinoma.

### Assessment of nutrient proportions

All case patients and control subjects were personally interviewed by professional interviewers from Statistics Sweden to provide data on background variables and various exposures, including a detailed food frequency questionnaire. The interviewers could not be blinded to the case/control status of the interviewees, but they were unaware of the study hypothesis and were urged to treat the cases and controls in a strictly equal manner. This food frequency questionnaire used was adopted from a validated standard questionnaire, which has been found to have high validity and reproducibility regarding assessment of dietary patterns [14], [15]. The information from the food frequency questionnaire was used to assess the relative proportions of the three main dietary components, i.e. carbohydrates, protein, and fat, of each study participant. The exposure data was based on a selected set of items regarding food and beverages, and we calculated the number of grams of carbohydrates, protein, and fat for each of the included items. To do this, we calculated the frequency of the consumption of each item and multiplied it by the average portion size. The portion sizes were assessed by the measures given by the Swedish National Food Administration. Thereafter, we calculated how many grams of carbohydrates, protein, and fat that each subject consumed per month by multiplying one hundred grams of the food or drink variable by how much each dietary item contained per 100 grams. The total consumption of carbohydrates, protein and fat was then transformed to energy intake in calories (1 gram carbohydrates  = 4000 calories, 1 gram protein = 9000 calories and 1 gram fat  = 7000 calories). The total monthly energy intake was then estimated for each participant and the proportion of carbohydrates, protein and fat of the monthly energy intake calculated. The proportions of intake of carbohydrates, protein, and fat were first grouped into quartiles based on the consumption among the control subjects, i.e. the cut-offs in 4 exposure groups of each of the 3 nutrient groups were based on 4 equally sized groups of control subjects. Finally, the relative distribution of all three macro nutrients was categorised into 6 groups. These 6 groups were created on the basis of a cut-off of the median consumption among the control subjects as high or low in each of the components: 1) a diet high in carbohydrates (≥48% of the total), low in protein (<37%) and low in fat (<24%) (reference category), 2) a diet high in carbohydrates (≥48%), high in protein (≥37%) and low in fat (<24%), 3) a diet high in carbohydrates (≥48%), low in protein (<37%) and high in fat (≥24%), 4) a diet low in carbohydrates (<48%), high in protein (≥37%) and low in fat (<24%), 5) a diet low in carbohydrates (<48%), low in protein (<37%) and high in fat (≥24%), and 6) a diet low in carbohydrates (<48%), high in protein (≥37%) and high in fat (≥24%).

### Assessment of cancer cases

All 195 Swedish hospital departments involved in the diagnosis or treatment of oesophageal cancer patients collaborated in the recruitment of cases. The six regional tumour registries enabled us to identify missing cases. There was a protocol for uniform documentation and classification of the tumours. At endoscopy, the distances between the oesophago-gastric junction (defined as the point where the proximal longitudinal mucosal folds begin in the stomach) and the upper and lower borders of the tumour, were measured. The protocol also prescribed that serial biopsy specimens should be taken every 2 cm from the proximal stomach, through the oesophago-gastric junction, in the oesophagus, until normal squamous-cell epithelium was reached. Additional specimens were to be obtained proximally, distally, and laterally to the tumour. Surgeons and pathologists completed standardised and detailed descriptions of the location of the cancer in operated cases. Moreover, 97% of all biopsies and surgical specimens were re-examined by one pathologist. An adenocarcinoma of the oesophago-gastric junction had to have its centre within 2 cm above, or 3 cm distal to the junction. If Barrett´s oesophagus was detected adjacent to the tumour, [16] it was classified as oesophageal, irrespective of its location.

### Statistical analysis

Unconditional logistic regression was used to analyse the exposures as continuous variables in relation to risk of the studied tumours and to analyse defined exposure categories as presented above, providing crude and adjusted odds ratios (OR) with 95% confidence intervals (95% CI). The cases in each cancer category were first compared with all controls, and in a second analysis, cases of adenocarcinoma of the oesophagus or oesophago-gastric junction were analysed jointly. In crude models, no adjustments were made, but the frequency matching provided similar distribution of age and sex. In the multivariable model, the results were adjusted for age, sex, reflux symptoms (heartburn or regurgitation at least once a week occurring at least 5 years before the interview, yes or no), body mass index (categorised into four groups from high to low by quartiles among the control subjects), tobacco smoking (never-, previous- or current smokers 2 years before the interview), alcohol use (grams of pure alcohol categorised into four groups from high to low by quartiles among the control subjects), years of formal education (>12 years, 7–12 years, or <7 years), and total energy intake (categorised in quartiles based on the consumption of the control participants).

## Results

### Study participants

The study included 189 cases of oesophageal adenocarcinoma (87% participation rate), 262 cases of oesophago-gastric adenocarcinoma (86% participation rate), 167 cases of oesophageal squamous cell carcinoma (73% participation rate), and 820 control subjects (73% participation rate). Characteristics of the participating case and control subjects are presented in Table 1. The median ages ranged from 66 to 69 years in the four groups of participants. There was an expected male predominance. Tobacco smoking and alcohol drinking were most common among the cases of oesophageal squamous cell carcinoma, while reflux and obesity were most common among the cases of oesophageal adenocarcinoma (Table 1).

**Table 1:** Characteristics of the 1,438 study participants.

	Oesophageal adenocarcinoma Number (%)	Oesophago-gastric junctional adenocarcinoma Number (%)	Oesophageal squamous cell carcinoma Number (%)	Control subjects Number (%)
**Number (% of eligible)**	189 (87%)	262 (83%)	167 (73%)	820 (73%)
**Median age**	69	66	67	68
**Males**	165 (87%)	223 (85%)	120 (72%)	679 (83%)
**Ever smokers** *	132 (70%)	219 (84%)	145 (87%)	495 (60%)
**Heavy alcohol drinkers** **	43 (23%)	76 (29%)	78 (47%)	178 (22%)
**Heartburn or regurgitation** **Yes** **No**	113 (60%) 76 (40%)	75 (29%) 187 (71%)	25 (15%) 142 (85%)	135 (16%) 685 (84%)
**Body mass index** *** ** <22 (low)** **2–24.9 (normal)** **25–30 (overweight)** **>30 (obesity)**	10 (5%) 68 (36%) 89 (47%) 22 (12%)	47 (18%) 100 (38%) 91 (35%) 24 (9%)	48 (29%) 67 (40%) 42 (25%) 10 (6%)	207 (25%) 366 (45%) 218 (27%) 25 (3%)
**Low educational level** ****	48 (23%)	43 (16%)	41 (25%)	182 (22%)
**Total energy intake 1^st^ quartile 2^nd^ quartile 3^rd^ quartile 4^th^ quartile**	52 (28%) 39 (21%) 54 (29%) 43 (23%)	63 (24%) 69 (26%) 64 (24%) 66 (25%)	46 (28%) 42 (25%) 36 (22%) 43 (26%)	205 (25%) 205 (25%) 205 (25%) 205 (25%)

* Tobacco smoking status including cigarette, cigar, and pipe smoking was assessed two years before the interview.

** High alcohol consumption was defined as more than 70 grams of pure alcohol per week.

*** Body mass index was assessed 20 years before interview, and calculated as body weight divided by the square of the height in meters (kg/m^2^).

**** Low educational level was defined as less than 7 years of formal education.

### Risk of oesophageal adenocarcinoma

High proportions of consumption of carbohydrates were followed by a possibly decreased risk of oesophageal adenocarcinoma (Table 2). The model with continuous exposure showed an adjusted OR of 0.77 (95% CI 0.58–1.03) per 10% increase in proportion of carbohydrates of total energy intake. Subjects in the highest quartile of the proportion of carbohydrates consumption were at a possibly decreased risk compared to those in the lowest quartile of consumption (adjusted OR 0.68, 95% CI 0.40–1.16). A correspondingly high proportion of consumption of protein did not influence this risk (adjusted OR 0.86, 95% CI 0.51–1.45). The highest quartile of proportion of fat intake indicated an increased risk of oesophageal adenocarcinoma compared to a low proportion of fat intake (adjusted OR 1.82, 95% CI 1.07–3.10), and a 10% increase in proportion of fat showed an adjusted OR of 1.65 (95% CI 1.07–2.55). Compared to a diet high in carbohydrates and low in both protein and fat, a diet low in carbohydrates and high in protein and fat (adjusted OR 1.46, CI 0.90–2.37), a diet low in carbohydrates, low in protein and high in fat (adjusted OR 1.68, CI 0.85–3.32), and a diet low in carbohydrates, high in protein and low in fats (adjusted OR 1.46, CI 0.71–3.00), were followed by a possibly increased risk of oesophageal adenocarcinoma, but the results were not statistically significant (Table 2).

**Table 2:** Association between proportion of monthly energy intake of the dietary macro nutrients carbohydrates, fats, and proteins and risk of oesophageal adenocarcinoma, expressed as odds ratios (OR) with 95% confidence intervals (CI).

		Oesophageal adenocarcinoma		
Dietary proportions	Number of controls (%)	Number of cases (%)	Crude OR (95%) CI	Adjusted* OR (95% CI)
**Carbohydrate Continuous (per 10% increase) Categories** ** **I (low) II III IV (high)**	****820 (100) 205 (25) 204 (25) 201 (24) 210 (26)	****188 (100) 62 (33) 51 (27) 33 (18) 42 (22)	0.74 (0.58–0.93) 1.00 (reference) 0.83 (0.54–1.26) 0.54 (0.34–0.86) 0.66 (0.43–1.02)	****0.77 (0.58–1.03) 1.00 (reference) 0.94 (0.57–1.55) 0.55 (0.32–0.97) 0.68 (0.40–1.16)
**Protein Continuous (per 10% increase) Categories** ** **I (low) II III IV (high)**	****820 (100) 201 (25)****204 (25)****207 (25)****208 (25)	****188 (100) 53 (28) 30 (16) 51 (27) 54 (29)	****1.27 (0.85–1.90) 1.00 (reference) 0.56 (0.34–0.91) 0.93 (0.61–1.44) 0.98 (0.64–1.51)	1.09 (0.68–1.76) 1.00 (reference) 0.59 (0.34–1.04) 1.14 (0.69–1.88) 0.86 (0.51–1.45)
**Fat Continuous (per 10% increase) Categories** ** **I (low) II III IV (high)**	****820 (100)****204 (25)****200 (24)****207 (25)****209 (26)	****188 (100)****37 (20)****41 (22)****46 (24)****64 (34)	****1.69 (1.16–2.44) 1.00 (reference) 1.13 (0.70–1.84) 1.23 (0.76–1.97) 1.69 (1.08–2.64)	****1.65 (1.07–2.55) 1.00 (reference) 1.36 (0.78–2.36) 1.21 (0.70–2.10) 1.82 (1.07–3.10)
**Distribution model** *** **: C-high, P-low, F-low C-high, P-high, F-low C-high, P-low, F-high C-low, P-high, F-low C-low, P-low, F-high C-low, P-high, F-high**	****259 (32) 83 (10) 69 (8) 62 (8) 77 (9) 270 (33)	****49 (26)****12 (6) 14 (7) 17 (9) 20 (11) 76 (40)	****1.00 (reference) 0.76 (0.39–1.51) 1.07 (0.56–2.06) 1.45 (0.78–2.69) 1.37 (0.77–2.45) 1.49 (1.00–2.21)	1.00 (reference) 0.93 (0.43–2.01) 0.86 (0.40–1.84) 1.46 (0.71–3.00) 1.68 (0.85–3.32) 1.46 (0.90–2.37)

* Adjusted for sex, age, reflux, BMI, smoking, alcohol consumption, education grade, and total energy intake.

** Categorised into quartiles of intake reported by the control subjects.

*** C-high = Carbohydrate proportion ≥48%, C-low = Carbohydrate proportion <48%, P-high = Protein proportion ≥37%, P-low = Protein proportion <37%, F-high = Fat proportion ≥24%, F-low = Fat proportion <24%.

### Risk of oesophago-gastric junctional adenocarcinoma

A high proportion of carbohydrate intake (highest quartile) was associated with a more than halved risk of oesophago-gastric junctional adenocarcinoma compared to a low proportion (adjusted OR 0.41, 95% CI 0.26–0.66) (Table 3). 10% increase in the proportion of carbohydrates showed an adjusted OR of 0.73 (95% CI 0.58–0.92). The proportions of protein consumption did not influence this risk. A high fat proportion intake increased the risk, and those who consumed protein in the highest quartile were at a 2-fold increased risk of oesophago-gastric junctional adenocarcinoma compared to those in the lowest quartile (adjusted OR 2.10, 95% CI 1.33–3.32), with a 10% increase corresponding to an OR of 1.62 (95% CI 1.14–2.30) in the model with continuous exposure. A diet with a low proportion of carbohydrates and a high proportion of both fat and protein increased the risk by over 50%, compared to a diet with a high proportion of carbohydrates and low proportions of protein and fat (adjusted OR 1.57, 95% CI 1.05–2.35) (Table 3).

**Table 3:** Association between proportion of monthly energy intake of the dietary macro nutrients carbohydrates, fats, and proteins and risk of oesophago-gastric junctional adenocarcinoma, expressed as odds ratios (OR) with 95% confidence intervals (CI).

		Oesophago-gastric junctional adenocarcinoma		
Dietary proportions	Number of controls (%)	Number of cases (%)	Crude OR (95% CI)	Adjusted* OR (95% CI)
**Carbohydrate Continuous (per 10% increase) Categories** ** **I (low) II III IV (high)**	****820 (100) 205 (25) 204 (25) 201 (24) 210 (26)	****262 (100) 94 (36) 67 (26) 60 (23) 41 (16)	****0.71 (0.57–0.87) 1.00 (reference) 0.72 (0.5–1.04) 0.65 (0.45–0.95) 0.43 (0.28–0.64)	****0.73 (0.58–0.92) 1.00 (reference) 0.73 (0.49–1.10) 0.67 (0.44–1.03) 0.41 (0.26–0.66)
**Protein Continuous (per 10% increase) Categories** ** **I (low) II III IV (high)**	****820 (100)****201 (25)****204 (25)****207 (25)****208 (25)	****262 (100) 59 (23) 54 (21) 65 (25) 84 (32)	1.46 (1.04–2.07) 1.00 (reference) 0.90 (0.59–1.37) 1.07 (0.72–1.60) 1.38 (0.94–2.02)	****1.32 (0.90–1.94) 1.00 (reference) 1.00 (0.64–1.55) 1.11 (0.73–1.70) 1.27 (0.83–1.96)
**Fat Continuous (per 10% increase) Categories** ** **I (low) II III IV (high)**	****820 (100) 204 (25) 200 (24) 207 (25) 209 (26)	****262 (100) 42 (16) 64 (24) 71 (27) 85 (32)	****1.67 (1.20–2.31) 1.00 (reference) 1.55 (1.01–2.40) 1.67 (1.09–2.56) 1.98 (1.30–3.00)	****1.62 (1.14–2.30) 1.00 (reference) 1.79 (1.13–2.84) 1.75 (1.11–2.76) 2.10 (1.33–3.32)
**Distribution model** *** **: C-high, P-low, F-low C-high, P-high, F-low C-high, P-low, F-high C-low, P-high, F-low C-low, P-low, F-high C-low, P-high, F-high**	****259 (32) 83 (10) 69 (8) 62 (8) 77 (9) 270 (33)	****60 (23) 19 (7) 22 (8) 27 (10) 31 (12) 103 (39)	****1.00 (reference) 0.99 (0.56–1.75) 1.38 (0.79–2.40) 1.88 (1.10–3.20) 1.74 (1.05–2.87) 1.65 (1.15–2.36)	****1.00 (reference) 0.89 (0.49–1.62) 1.32 (0.73–2.38) 1.80 (1.01–3.21) 1.75 (1.02–3.01) 1.57 (1.05–2.35)

* Adjusted for sex, age, reflux, BMI, smoking, alcohol consumption, education grade, and total energy intake.

** Categorised into quartiles of intake reported by the control subjects.

*** C-high = Carbohydrate proportion ≥48%, C-low = Carbohydrate proportion <48%, P-high = Protein proportion ≥37%, P-low = Protein proportion <37%, F-high = Fat proportion ≥24%, F-low = Fat proportion <24%.

### Risk of oesophageal or oesophago-gastric junctional adenocarcinoma

Since oesophageal and oesophago-gastric junctional adenocarcinomas are closely located and share many characteristics and aetiological factors, these tumours were combined in an analysis to increase statistical power. A high proportion of carbohydrates decreased the risk of these tumours (adjusted OR 0.50, CI 0.34–0.73), while a high proportion of fat increased the risk nearly 2-fold (adjusted OR 1.96, CI 1.34–2.87) (Table 4). A high proportion of protein seemed not to influence the risk of oesophageal or oesophago-gastric junctional adenocarcinoma (adjusted OR 1.08, CI 0.75–1.56). These results were supported by the model with continuous exposure (Table 4). Compared to a diet high in carbohydrates and low in both protein and fat, a dietary distribution low in carbohydrates, low in protein and high in fat (adjusted OR 1.73, CI 1.09–2.75), a diet low in carbohydrates, high in protein and low in fat (adjusted OR 1.69, CI 1.03–2.79), and a diet low in carbohydrates, high in protein and high in fat (adjusted OR 1.51, CI 1.07–2.12) increased the risk of these tumours (Table 4).

**Table 4:** Association between proportion of monthly energy intake of the dietary macro nutrients carbohydrates, fats, and proteins and risk of oesophageal or oesophago-gastric junctional adenocarcinoma, expressed as odds ratios (OR) with 95% confidence intervals (CI).

		Oesophageal or oesophago-gastric junctional adenocarcinoma		
Dietary proportions	Number of controls (%)	Number of cases (%)	Crude OR (95%) CI	Adjusted* OR (95% CI)
**Carbohydrate Continuous (per 10% increase) Categories** ** **I (low) II III IV (high)**	****820 (100) 205 (25) 204 (25) 201 (24) 210 (26)	****450 (100) 156 (35) 118 (27) 93 (21) 83 (19)	****0.72 (0.61–0.86) 1.00 (reference) 0.76 (0.56–1.03) 0.61 (0.44–0.84) 0.52 (0.37–0.72)	****0.74 (0.61–0.91) 1.00 (reference) 0.78 (0.55–1.11) 0.63 (0.43–0.92) 0.50 (0.34–0.73)
**Protein Continuous (per 10% increase) Categories** ** **I (low) II III IV (high)**	****820 (100) 201 (25) 204 (25) 207 (25) 208 (25)	****450 (100) 112 (26) 84 (19) 116 (26) 138 (31)	****1.37 (1.03–1.83) 1.00 (reference) 0.74 (0.52–1.04) 1.01 (0.73–1.39) 1.19 (0.87–1.63)	****1.24 (0.89–1.72) 1.00 (reference) 0.81 (0.56–1.18) 1.06 (0.74–1.51) 1.08 (0.75–1.56)
**Fat Continuous (per 10% increase) Categories** ** **I (low) II III IV (high)**	****820 (100) 204 (25) 200 (24) 207 (25) 209 (26)	****450 (100) 79 (18) 105 (23) 117 (26) 149 (33)	****1.67 (1.28–2.19) 1.00 (reference) 1.36 (0.95–1.93) 1.46 (1.03–2.06) 1.84 (1.32–2.57)	****1.64 (1.21–2.22) 1.00 (reference) 1.62 (1.10–2.39) 1.58 (1.08–2.32) 1.96 (1.34–2.87)
**Distribution model** *** **: C-high, P-low, F-low C-high, P-high, F-low C-high, P-low, F-high C-low, P-high, F-low C-low, P-low, F-high C-low, P-high, F-high**	****259 (32) 83 (10) 69 (8) 62 (8) 77 (9) 270 (33)	****109 (25) 31 (7) 36 (8) 44 (10) 51 (12) 179 (40)	****1.00 (reference) 0.89 (0.56–1.42) 1.24 (0.78–1.97) 1.69 (1.08–2.64) 1.57 (1.04–2.39) 1.58 (1.18–2.11)	****1.00 (reference) 0.87 (0.52–1.44) 1.19 (0.71–1.98) 1.69 (1.03–2.79) 1.73 (1.09–2.75) 1.51 (1.07–2.12)

* Adjusted for sex, age, reflux, BMI, smoking, alcohol consumption, education grade, and total energy intake.

** Categorised into quartiles of intake reported by the control subjects.

*** C-high = Carbohydrate proportion ≥48%, C-low = Carbohydrate proportion <48%, P-high = Protein proportion ≥37%, P-low = Protein proportion <37%, F-high = Fat proportion ≥24%, F-low = Fat proportion <24%.

### Risk of oesophageal squamous cell carcinoma

The proportions of consumption of carbohydrates did not influence the risk of oesophageal squamous cell carcinoma (Table 5). Participants in the highest proportion quartile of carbohydrates had no decreased risk of this cancer compared to those in the lowest quartile (adjusted OR 1.05, 95% CI 0.61–1.80). Higher proportions of protein consumption were followed by a tendency towards an increased risk, but not statistically significantly (Table 5). The highest quartile of proportion of fat consumption indicated a possibly decreased risk of oesophageal squamous cell carcinoma compared to the lowest quartile (adjusted OR 0.73, 95% CI 0.42–1.27),and a diet distribution low in carbohydrates, low in protein, and high in fat entailed a decreased risk compared to a diet high in carbohydrates, low in fat and low in protein (adjusted OR 0.32, 95% CI 0.12–0.81). No statistically significant associations were found when the macronutrients were analysed as continuous variables (Table 5).

**Table 5:** Association between proportion of monthly energy intake of the dietary macro nutrients carbohydrates, fats, and proteins and risk of oesophageal squamous cell carcinoma, expressed as odds ratios (OR) with 95% confidence intervals (CI).

		Oesophageal squamous cell carcinoma		
Dietary proportions	Number of controls (%)	Number of cases (%)	Crude OR (95% CI)	Adjusted* OR (95% CI)
**Carbohydrate Continuous (per 10% increase) Categories** ** **I (low) II III IV (high)**	****820 (100) 205 (25) 204 (25) 201 (24) 210 (26)	****167 (100) 43 (26) 43 (26) 35 (21) 46 (28)	****1.04 (0.81–1.33) 1.00 (reference) 1.00 (0.63–1.60) 0.83 (0.51–1.35) 1.04 (0.66–1.65)	****1.07 (0.81–1.42) 1.00 (reference) 1.00 (0.58–1.73) 0.86 (0.49–1.52) 1.05 (0.61–1.80)
**Protein Continuous (per 10% increase) Categories** ** **I (low) II III IV (high)**	****820 (100) 201 (25) 204 (25) 207 (25) 208 (25)	****167 (100) 47 (28) 28 (17) 41 (25) 51 (31)	****0.99 (0.65–1.51) 1.00 (reference) 0.59 (0.35–0.97) 0.85 (0.53–1.34) 1.05 (0.67–1.63)	****1.09 (0.67–1.76) 1.00 (reference) 0.74 (0.42–1.29) 0.93 (0.55–1.56) 1.15 (0.68–1.94)
**Fat Continuous (per 10% increase) Categories** ** **I (low) II III IV (high)**	****820 (100) 204 (25) 200 (24) 207 (25) 209 (26)	****167 (100) 42 (25) 42 (25) 46 (28) 37 (22)	****0.91 (0.62–1.36) 1.00 (reference) 1.02 (0.64–1.63) 1.08 (0.68–1.71) 0.86 (0.53–1.39)	****0.78 (0.50–1.22) 1.00 (reference) 0.97 (0.57–1.65) 1.01 (0.60–1.69) 0.73 (0.42–1.27)
**Distribution model** *** **: C-high, P-low, F-low C-high, P-high, F-low C-high, P-low, F-high C-low, P-high, F-low C-low, P-low, F-high C-low, P-high, F-high**	****259 (32) 83 (10) 69 (8) 62 (8) 77 (9) 270 (33)	****57 (34) 12 (7) 12 (7) 15 (9) 6 (49) 65 (39)	****1.00 (reference) 0.66 (0.34–1.28) 0.79 (0.40–1.55) 1.10 (0.58–2.07) 0.35 (0.15–0.85) 1.09 (0.74–1.62)	****1.00 (reference) 0.58 (0.28–1.21) 0.79 (0.37–1.67) 1.33 (0.64–2.76) 0.32 (0.12–0.81) 1.02 (0.64–1.62)

* Adjusted for sex, age, reflux, BMI, smoking, alcohol consumption, education grade, and total energy intake.

** Categorised into quartiles of intake reported by the control subjects.

*** C-high = Carbohydrate proportion ≥48%, C-low = Carbohydrate proportion <48%, P-high = Protein proportion ≥37%, P-low = Protein proportion <37%, F-high = Fat proportion ≥24%, F-low = Fat proportion <24%.

Generally, the crude ORs were similar to the fully adjusted ORs (Tables 2, 3, 4, 5).

## Discussion

This study indicates that a high proportion of carbohydrate consumption of the total energy intake decreases the risk of oesophageal and oesophago-gastric junctional adenocarcinoma, whereas a high proportion of fat in the diet increases the risk of these tumours, and proportions of protein consumption do not influence this risk. These nutrient proportions did not clearly influence the risk of oesophageal squamous cell carcinoma.

Strengths of the study include the population-based design with high participation rates, the thorough and uniform classification of all tumours, the ability to adjust the results for all established risk factors, and the complete and rapid case ascertainment, which enabled personal interviews with all participants. Among weaknesses is the misclassification of the exposure, which might be increased by the fact that we asked about dietary habits 20 years prior to interview. This method of assessment of dietary items 20 years earlier has, however, been validated with good results, showing that such assessment captures a combination of previous and current dietary habits [17]. Moreover, the exposure misclassification should be non-differential, i.e., similar between cases and controls, and thus the true risk estimates are probably underestimated as a result of dilution [18]. The assessment of nutrient proportions based on food frequency questionnaires might be a rough method prone to misclassification, but it is still the most validated and used measure of such exposures. Another concern is whether the assessment of proportions in relation to risk of oesophageal cancers actually measures the influence of proportions, or whether it is just an effect of specific carcinogenic dietary items within these groups of nutrients. Nevertheless, the diverging results for adenocarcinoma and squamous cell carcinoma indicate a role of proportions. These diverging findings also indicate that recall bias might not be a major issue, which is otherwise a threat to case-control studies with data collected from interviews. Finally, chance error cannot be excluded. There were a relatively small number of cases in each tumour group, which limited the precision. Moreover, the many comparisons conducted might introduce multiple testing errors.

Recent studies have provided further evidence that fruit and vegetable intake is associated with decreased risk of oesophageal and oesophago-gastric junctional adenocarcinoma and oesophageal squamous cell carcinoma, whereas meat and nitrate decreases the risk of these tumours [5]–[8], [19], [20]. A diet rich in foods of animal origin and poor in foods containing vitamins and fibre increases the risk of oesophageal cancer in general [5]. Moreover, a high-fat diet is associated with increased risk of both oesophageal cancer and junctional adenocarcinoma. Higher intake of meat, particularly red meat, is associated with an increased risk of oesophageal adenocarcinoma, while higher intake of meats such as poultry, and high-fat diary is associated with increased risk of junctional adenocarcinoma [19]. While previous studies have typically focused on the food items themselves or on other dietary patterns, this study addressed the role of proportions of energy intake between the macro nutrients carbohydrates, fats, and proteins in relation to oesophageal cancers. Most previous studies have found similar patterns of association between dietary factors and adenocarcinoma and squamous cell carcinoma of the oesophagus, but the differences in patterns found between the histological types in this study of macro nutrient proportions seem to indicate separate dietary aetiologies. The fat proportion in particular seemed to influence the risk of adenocarcinoma and squamous cell carcinoma of the oesophagus in opposite directions.

The oesophageal cancer specific mechanisms that might explain the findings of the present study can only be speculated upon. A high fat proportion might be followed by an increased prevalence of obesity, which in turn entails an increased risk of adenocarcinoma of the oesophagus and oesophago-gastric junction, but the results of the present study were thoroughly adjusted for the influence of BMI. Residual confounding by BMI cannot be entirely excluded, but alternative mechanisms should be considered. A high fat proportion diet might cause a slower passage of food through the stomach, i.e. inhibition of gastric emptying by intestinal peptides, [21] which in turn might result in an increased occurrence of gastro-oesophageal reflux. Although reflux symptoms were adjusted for in the analysis, an increased occurrence of asymptomatic (physiological) reflux cannot be dismissed as an explanation for the finding. The lack of any increased risk of oesophageal squamous cell carcinoma among a proportionally high-fat dietary pattern would support this hypothesised mechanism, since this cancer is not associated with reflux. The seemingly beneficial effects of a high dietary carbohydrate proportion specific to oesophageal and junctional adenocarcinoma would probably not be through high consumption of fruit and vegetables, since such consumption is at least as protective for squamous cell carcinoma of the oesophagus. A high proportion of carbohydrates might simply reflect a low proportion of fat, which in turn seems beneficial.

In conclusion, this population-based and nationwide Swedish study indicates that a high proportion of consumption of carbohydrates decreases the risk for oesophageal and oesophago-gastric junctional adenocarcinoma, while a high proportion of consumption of fat increases the risk and protein proportions did not influence this risk. Macro nutrient proportions did not much influence the risk of oesophageal squamous cell carcinoma. These differences indicate some separate dietary aetiology for the main histological types of oesophageal cancer.
